# Enhanced H_2_S Gas-Sensing Performance of Zn_2_SnO_4_ Lamellar Micro-Spheres

**DOI:** 10.3389/fchem.2018.00165

**Published:** 2018-05-14

**Authors:** Ting-Ting Xu, Ying-Ming Xu, Xian-Fa Zhang, Zhao-Peng Deng, Li-Hua Huo, Shan Gao

**Affiliations:** Key Laboratory of Functional Inorganic Material Chemistry, Ministry of Education, School of Chemistry and Materials Science, Heilongjiang University, Harbin, China

**Keywords:** Zn_2_SnO_4_, H_2_S gas, ppb-level, lamellar micro-spheres, sensitivity

## Introduction

With the rapid development of industry, the discharge of sulfide gas has been increasing in recent decades, which results in severe air pollution. H_2_S, as the typical representation of sulfide, is a harmful and toxic acidic gas and widely used in various industries. Even at low concentrations, it can cause hypoxia and seriously threatens the safety of human. When the concentration reaches 1 mg/L (659 ppm) or higher, inflammation or death will occur. According to the U. S. Scientific Advisory Board on Toxic Air Pollutants, the acceptable concentration of H_2_S in the environment is less than 83 ppb (North Carolina Department of Environment and Natural Resources, 2003^1^). Therefore, it is necessary to fabricate gas sensors which can detect ppb-level H_2_S in time to reduce the environmental pollution and harm to human.

Zinc stannate (Zn_2_SnO_4_) is a typical n-type ternary semiconductor, and has been employed as important multifunctional material in the fields of photocatalytic activity (Das et al., 2017), solar cells (Li et al., 2015), lithium ion batteries (Lim et al., 2016), and so forth. Especially, owing to the excellent gas-sensing performance of ZnO and SnO_2_ (Sukunta et al., 2017; Zhu and Zeng, 2017; Zhu et al., 2018), the application of Zn_2_SnO_4_ in the field of gas sensors has attracted extensive attention (An et al., 2015; Zhao et al., 2016; Yang et al., 2017). Up to now, only one case has concerned on the detection of H_2_S with Zn_2_SnO_4_ hollow octahedron (Ma et al., 2012), which showed the response to H_2_S with the detection limit being 1 ppm at 260°C. Meanwhile, such reported Zn_2_SnO_4_ sensor presents poor selectivity. Apparently, the sensor cannot satisfy the need of practical application for the detection of ppb-level H_2_S, especially in a complex environment involving other interfering gases due to its poor selectivity and higher working temperature. In view of the fact that gas-sensing performance of materials is highly dependent on their micro-structure and surface state (Yu et al., 2017; Zhang et al., 2018), therefore, it would be a meaningful work to prepare Zn_2_SnO_4_ with novel morphology to further decrease the working temperature and improve the selectivity and stability, thus performing the detection of ppb-level H_2_S.

In this work, Zn_2_SnO_4_ lamellar micro-spheres have been synthesized by a facile ethylenediamine-assisted hydrothermal method followed by calcining at 600°C. The diameter of micro-spheres is ~1 μm and they are composed of nanosheets with thickness of ~85 nm. The sensor fabricated from the micro-spheres shows good response and selectivity to H_2_S at 170°C, and the lowest detection limit is down to 50 ppb. Moreover, it shows good linear relationship in the range of ppb (50–1000 ppb) and ppm (3–50 ppm) level. Meanwhile, the gas-sensing mechanism is also investigated.

## Experimental

### Preparation of Zn_2_SnO_4_ lamellar micro-spheres

All the reagent were analytical grade (AR) and used as received without supplementary purification. Zn(CH_3_COO)_2_·2H_2_O (1.2 mmol) and SnCl_4_·5H_2_O (0.6 mmol) were dissolved in the mixture of 20 mL deionized water and 10 mL ethylenediamine. After stirring for 30 min, 7.2 mmol NaOH was added to the above system followed by further stirring for 1 h. Then, the above turbid solution was transferred into a 50 mL Teflon-lined stainless steel autoclave and kept at 200°C for 24 h. After cooling to room temperature, the precipitate was centrifuged and washed with deionized water and ethanol for several times. White products were obtained after drying at 60°C for 12 h, which was then calcined at 600°C for 2 h in air atmosphere to obtain the Zn_2_SnO_4_ lamellar micro-spheres.

The characterization, sensor fabrication and measurement were listed in the Supporting Information.

## Results and discussion

**Figure 1:**
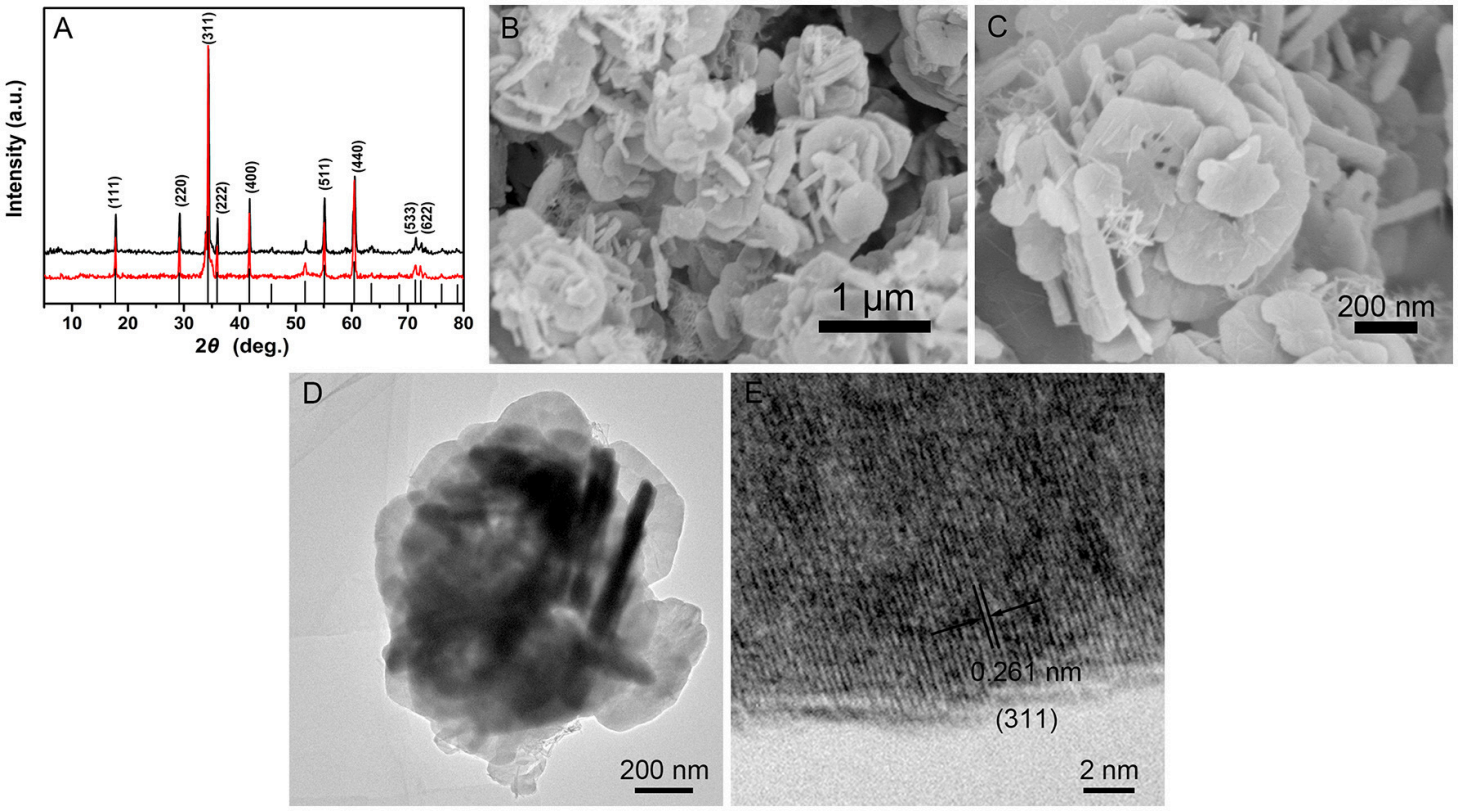
**(A)** XRD patterns of the precursor and the calcined product. **(B,C)** SEM images of the calcined product. **(D,E)** TEM and HRTEM images of the calcined product.

The FT-IR spectra of the precursor and calcined product were illustrated in Figure S1a. After calcination, the corresponding vibrations of acetate disappear. The spectrum of calcined product exhibits one peak at 3417 cm^−1^, corresponding to the stretching vibration of water molecules. The Sn-O and Zn-O vibrations at 572 and 431 cm^−1^ in this spectrum are slightly enhanced, which indicates that the calcined product has excellent crystallinity. The TG curve of the precursor (Figure S1b) shows that the marked weight loss of 6.27% between 30 and 600°C can be attributed to the loss of water molecules and small amount of acetates. After 600°C, no obvious weight loss occurs with the increase of temperature. Therefore, the precursor is calcined at 600°C for 2 h. The XRD patterns of the precursor and calcined product are also conducted to confirm their identity and phase purity. As illustrated in Figure 1A, all the diffraction peaks in both precursor and calcined product are consistent with standard cubic Zn_2_SnO_4_ (JCPDS Card No. 24-1470). In comparison with the precursor, the calcined product presents stronger diffraction peaks and better consistency. It shows that the calcined product has better crystallinity. The surface components of the calcined product characterized by XPS spectrum indicate that the final product involves Zn, Sn and O elements (Figure S2).

As shown in Figure 1B, the micro-spheres of the precursor with diameter of ~1.5 μm are composed of smooth nanosheets with thickness of ~130 nm. After being calcined at 600°C in air, the calcined products maintain the morphology of precursor, and the diameter of the micro-spheres is slightly decreased from ~1.5 to ~1 μm (Figure 1C and Figure S3). The thickness of the nanosheets is also sharply reduced to ~85 nm. The results shown in Figure S4 reveal that the three elements are distributed very homogeneously in the micro-spheres. Figure 1D shows TEM image for the Zn_2_SnO_4_ lamellar micro-sphere which presents a clear contrast between the dark and the pale parts, revealing that the micro-spheres are further assembled by nanosheets. The high resolution TEM (HRTEM) image in Figure 1E exhibits the clear parallel fringes with *d*-spacing of 0.261 nm for nanosheet, which corresponds to the (311) lattice planes of cubic Zn_2_SnO_4_. Meanwhile, some micro-pores appears on the surface of the nanosheets, which implies better gas performance of the lamellar micro-spheres.

**Figure 2:**
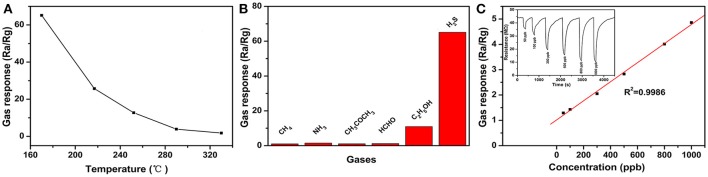
**(A)** The response of Zn_2_SnO_4_ lamellar micro-spheres sensor toward 100 ppm of H_2_S at different working temperatures. **(B)** The response of Zn_2_SnO_4_ hierarchical microspheres sensor toward 100 ppm of different testing gases at 170°C. **(C)** The relationship between the responses of Zn_2_SnO_4_ lamellar micro-spheres sensor and ppb-level concentrations of H_2_S (Inset: the response-recovery characteristics of the sensor to different concentrations of H_2_S at 170°C).

Figure 2A is the response of Zn_2_SnO_4_ lamellar micro-spheres sensor toward 100 ppm of H_2_S at different working temperatures. As can be seen, the response of sensor to H_2_S decreases with the increase of working temperature. This is mainly because the adsorbed H_2_S gas molecules escaped from the surface of materials easily before the reaction taking place at high operating temperature so as to a poor response as well. In other word, such commonly observed phenomenon in resistive sensors is mainly ascribed to the fact that the desorption plays dominant role at higher temperature which leads to the decrease in sensitivity. It shows that the best working temperature for the Zn_2_SnO_4_ sensor is 170°C, which is 90°C lower than that of Zn_2_SnO_4_ hollow octahedron (Ma et al., 2012). Thus, the following tests were carried out at 170°C. Figure 2B is the response of the sensor toward 100 ppm of CH_4_, NH_3_, CH_3_COCH_3_, HCHO, C_2_H_5_OH and H_2_S at 170°C. The responses to the above gases are 1.04, 1.49, 1.08, 1.2, 10.95, 65.13, respectively, which show that the sensor presents the best response to H_2_S than other gases. The selectivity coefficients (K_AB_) of H_2_S to other gases are 62.63, 43.71, 60.31, 54.27, 5.94, respectively, indicating excellent selectivity of the sensor to H_2_S. Such highly H_2_S selectivity can be attributed to the reactivities of the reducing test gases. The bond energy of 381 kJ/mol for H-SH in H_2_S (Liu et al., 2009) is smaller than other inorganic gases and most of organic gases, so that H-SH bond can be easily broken to participate in the reaction with gas sensor during chemical adsorption. Given all that, the sensor has good response and selectivity to H_2_S at 170°C.

The relationship between the responses of the sensor and ppb-level concentrations of H_2_S and the response-recovery characteristics of the sensor to different concentrations of H_2_S at 170°C are shown in Figure 2C. The gas response of the sensor increases in a good linear relationship from 50 to 1000 ppb (*R*^2^ = 0.9986), indicating that the detection limit of the sensor is 50 ppb (response of 1.29) to H_2_S. In contrast to the reported Zn_2_SnO_4_ octahedron sensor (Ma et al., 2012), the present sensor displays lower working temperature and ppb-level detection limit. Even compared with other preferable ternary metal oxides, such as La_2_NiO_4_ particles (500°C, 20 ppb, Hao et al., 2018), K_2_W_4_O_13_ nano-wires (300°C, 0.3 ppm, Supothina et al., 2014), Fe_2_(MoO_4_)_3_ nano-particles and micro-spheres (300°C, 1 ppm, Liang et al., 2016), the present material still shows better sensing performance toward H_2_S. To the best of our knowledge, the detection limit of Zn_2_SnO_4_ lamellar micro-spheres sensor is only slightly higher than that of flower-like Bi_2_MoO_6_ (Cui et al., 2017) which has the lowest detection limit of 0.1 ppb to H_2_S. The response of the sensor in the range from 3 to 50 ppm also increases in a good linear relationship (*R*^2^ = 0.9927) (Figure S5). The responses of the sensor to 10 ppm H_2_S during 5 consecutive tests at 170°C is shown in Figure S6a. It shows that the sensor keeps its initial response amplitude after 5 cycles. The sensor measurement also maintains initial response to 10 ppm H_2_S with the standard error of 4.9% after 60 days (Figure S6b). These results indicate that this sensor has a satisfactory reproducibility and stability. Meanwhile, the influence of relative humidity on the sensor was considered at 170°C (Figure S7). It can be seen from Figure S7 that the sensor response has a small change from 1.2 to 1.4 in different humidity environment, suggesting that the influence of humidity on the sensor can be neglected at such temperature. Therefore, the present Zn_2_SnO_4_ lamellar micro-spheres sensor could be utilized as promising material for detecting ppb-level H_2_S.

It is considered that the gas sensing property of semiconductor oxides is related to the surface adsorption oxygen, therefore, the chemical state changes of each element in the Zn_2_SnO_4_ sensor were analyzed before and after the sensor contacting with H_2_S at 170°C. The results indicate that there is no change in the spectra of the Zn 2p and Sn 3d (Figure S8). As shown in Figures S9a,b, the O 1s spectra of Zn_2_SnO_4_ could be deconvoluted into three peaks at 531.9/531.8, 530.8/530.7, 529.4/529.3, corresponding to hydroxyl oxygen, surface adsorbed oxygen and lattice oxygen, respectively. The percentage of surface adsorbed oxygen drops from 30.04 to 27.01% after the sensor contacting with H_2_S. The obvious decreasing of the adsorbed oxygen indicates that H_2_S participates in redox reaction with the surface adsorbed oxygen. When the Zn_2_SnO_4_ sensor is exposed to H_2_S at 170°C, the S 2p XPS spectrum displays three peaks at 168.9, 163.2 and 161.5 eV that correspond to SO_2_ and sulfide (S 2p_3/2_ and S 2p_1/2_) (Figure S9c), respectively. The appearance of the three peaks indicates that H_2_S is oxidized to SO_2_. Combined with previous reports (Yu et al., 2015), the redox reaction engenders between H_2_S and the surface adsorbed oxygen, and generates the product of SO_2_.

Based on the aforementioned results, the gas sensing mechanism is speculated as follows: the resistance changes of the gas sensor are observed before and after contacting with H_2_S. When the Zn_2_SnO_4_ sensor is exposed in air, oxygen molecules which is adsorbed on the surface of the sensor (Equation 1) captures electrons in conduction band of Zn_2_SnO_4_ to form of O^−^ at 170°C (Equation 2), and engender electron depletion layer. Then, after the sensor contacting with the H_2_S, the H_2_S is oxidized by the O^−^ on the surface of sensor and releases electrons back into the conduction band (Equations 3, 4), resulting in an increase in surface electrons and conductivity, and a decrease in resistance.

(1) $$\begin{array}{r} {\text{O}}_{2}\left(\text{gas}\right)\to {\text{O}}_{2}\left(\text{ads}\right) \end{array}$$

(2) $$\begin{array}{r} {\text{O}}_{2}\left(\text{ads}\right)+2{\text{e}}^{-}\to 2{\text{O}}^{-}\left(420-670\text{K}\right) \end{array}$$

(3) $$\begin{array}{r} {\text{H}}_{2}\text{S}\left(\text{gas}\right)\to {\text{H}}_{2}\text{S}\left(\text{ads}\right) \end{array}$$

(4) $$\begin{array}{r} {\text{H}}_{2}\text{S}\left(\text{ads}\right)+3{\text{O}}^{-}\left(\text{ads}\right)\to \text{S}{\text{O}}_{2}+{\text{H}}_{2}\text{O}+3{\text{e}}^{-}\left(443\text{K}\right) \end{array}$$

## Conclusion

In conclusion, a facile ethylenediamine-assisted hydrothermal method followed by calcining at 600°C led to the formation of Zn_2_SnO_4_ lamellar micro-spheres which comprise of nanosheets with thickness of ~85 nm. Such sensor exhibits excellent selectivity, sensitivity, humidity resistance and stability to H_2_S at working temperature 170°C. The responses of the sensor, increased with the increasing concentrations in the range of 50–1000 ppb and 3–50 ppm exhibit good relationships with the detection limit of 50 ppb. These results indicate that the Zn_2_SnO_4_ lamellar micro-spheres could be utilized as promising sensor material for detecting ppb-level H_2_S.

## Author contributions

T-TX performed the experiments and analyzed the data with the help from Y-MX and X-FZ. Z-PD wrote the manuscript with input from all authors. L-HH and SG conceived the study. All authors read and approved the manuscript.

### Conflict of interest statement

The authors declare that the research was conducted in the absence of any commercial or financial relationships that could be construed as a potential conflict of interest.

## Footnotes

^1^http://daq.state.nc.us/toxics/studies/H_2_S/ (accessed 2003).
