# Supplementary material for: Enhanced H2S Gas-Sensing Performance of Zn2SnO4 Lamellar Micro-Spheres
Source: Front Chem. 2018 May 14;6:165. doi: 10.3389/fchem.2018.00165 (PMC5960710; doi:10.3389/fchem.2018.00165)
Supplement: Supplementary file 1 [file Presentation_1.PDF]

Supplementary Material

Enhanced H_2S Gas-Sensing Performance of Zn_2SnO_4 Lamellar Micro-spheres

Ting-Ting Xu, Ying-Ming Xu, Xian-Fa Zhang, Zhao-Peng Deng*, Li-Hua Huo, Shan Gao*

Key Laboratory of Functional Inorganic Material Chemistry, Ministry of Education, School of Chemistry and Materials Science, Heilongjiang University, Harbin 150080, People's Republic of China.

*Correspondence: dengzhaopeng@hlju.edu.cn (Z. D.); shangao67@yahoo.com (S. G.)

Characterization

The phase and crystallinity of the product were performed by powder X-ray diffraction (PXRD, D8-Advance, Bruker,) with Cu-Kα radiation (λ = 0.154056 nm). The morphology and microstructure of the product were characterized by scanning electron microscopy (SEM, S-4800, Hitachi). The surface chemistry of the product was analyzed by X-ray photoelectron spectrometer (XPS, ULTRA AXIS DLD) and the binding energy values were corrected by referencing the C 1s line to 284.6 eV. Thermogravimetric (TG) analysis was characterized by a thermogravimetric analyzer (PERKIN ELMER, TG/DTA6300) from 30 to 900 ^{o}C at a heating rate of 5 ^{o}C min^{-1} in air atmosphere. Fourier transform infrared spectra (FT-IR) of the product were recorded with KBr pellets using a FT-IR spectrometer (Bruker Equinox, 55) with a mercury-cadmium-telluride (MCT) detector.

Sensor fabrication and measurement

The thick sensing film of Zn_2SnO_4 was obtained by coating the paste prepared from the mixing of a few drop of terpineol with appropriate Zn_2SnO_4 on the surface of an Al_2O_3 microtube bearing two Au electrodes and four Pt wires. Then, it was dried at 80 ^{o}C and calcinated at 400 ^{o}C for 1 h in air. After above process, a Ni-Cr heating wire was inserted into the tube as heater. The sensor was aged at 170 ^{o}C for 72 h before the gas sensing measurement to insure the stability and repeatability.

The gas responses of sensors were obtained by the JF02F gas sensing measurement system (Sino-platinum metal, China). In order to control the gas concentration, different amounts of test gas were separately added into the container. The test principle in present work was carried out according to that in previous report (Sui et al., 2015). The temperature and relative humidity of the environment were in the range of 15-18 °C and 20-30 RH%. The response of sensor (S) is defined as S = Ra/Rg (Ra is the resistance in the air and Rg is the resistance in the test gas). The selectivity coefficient (K_{AB}) of A gas to B gas is defined as S_A/S_B, where S_A and S_B are the responses of sensors measured in hydrogen sulfide (H_2S) gas and other gases (methane, ammonia, acetone, formaldehyde, and ethanol in present study). The response time is defined as the time that the sensor resistance changes to 100 s when the sensor exposed to the target gases.

The humidity sensing properties of sensors were also studied with different saturated salt solutions such as LiCl (11 RH%), $MgCl_2$ (33 RH%), $Mg(NO_3)_2$ (54 RH%), NaCl (75 RH%) and KNO_3 (94 RH%). Every saturated salt solution was placed for at least 24 h before test. The humidity response test of sensor was measured at 170 °C.

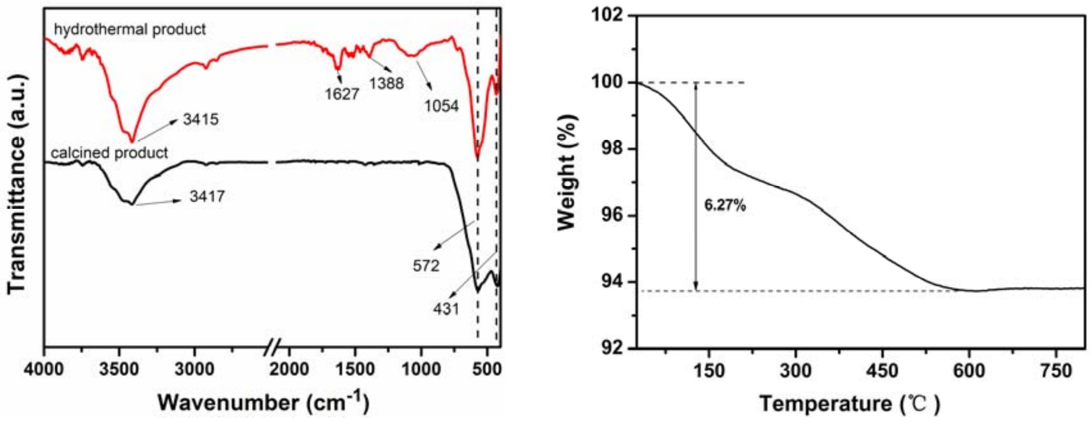

FIGURE S1 FT-IR spectra of hydrothermal product and calcined product (a) and the TG curve of the hydrothermal product (b).

From Figure S1a, the peak at 3415 cm^{-1} in the spectrum of precursor can be assigned to the vibration of water molecules. The weak peaks observed at 1627 and 1388 cm^{-1} are ascribed to the anti-symmetric and symmetric stretching vibrations of $–COO^-$(acetate), which indicates the existence of small amount of acetates involved in the precursor. The two strong peaks at 572 and 431 cm^{-1} are attributed to the characteristic vibrations of Sn-O and Zn-O.

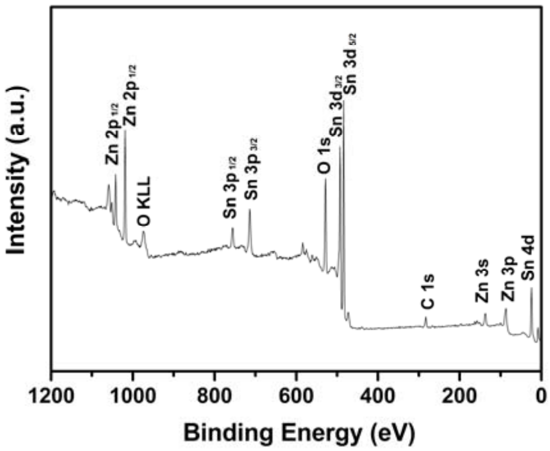

FIGURE S2 XPS spectrum of full survey of the calcined product.

The XPS spectrum of full survey of the calcined product is shown in Figure S2. The survey spectrum involves distinct peaks at 530.3 (O 1s), 1045.5 (Zn $2p_{1/2}$), 1021.2 (Zn $2p_{3/2}$), 139.9 (Zn 3s), 89.7 (Zn 3p), 757.4 (Sn $3p_{1/2}$), 717.5 (Sn $3p_{3/2}$), 494.9 (Sn $3d_{3/2}$), 486.5 (Sn $3d_{5/2}$), 25.5 (Sn 4d), which indicate that the final product involves Zn, Sn and O elements. The bonding energy difference between Sn $3d_{3/2}$ and Sn $3d_{5/2}$ peaks of 8.4 eV is consistent with the value of reported 3D hierarchical SnO_2, which is ascribed to the splitting of the spin orbit, and demonstrates that the valence of the tin element is Sn^{4+} rather than Sn^{2+} ion (Wang, Cheng et al., 2015). The results of the XPS spectrum further indicate that the calcined product is pure cubic Zn_2SnO_4 phase.

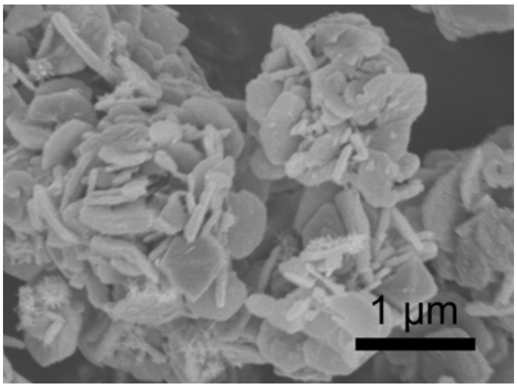

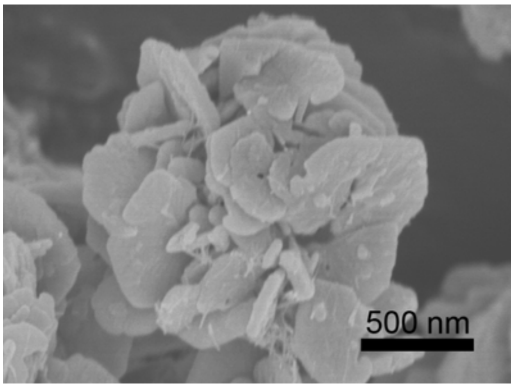

(a) (b)

FIGURE S3 (a, b) SEM images of the precursor.

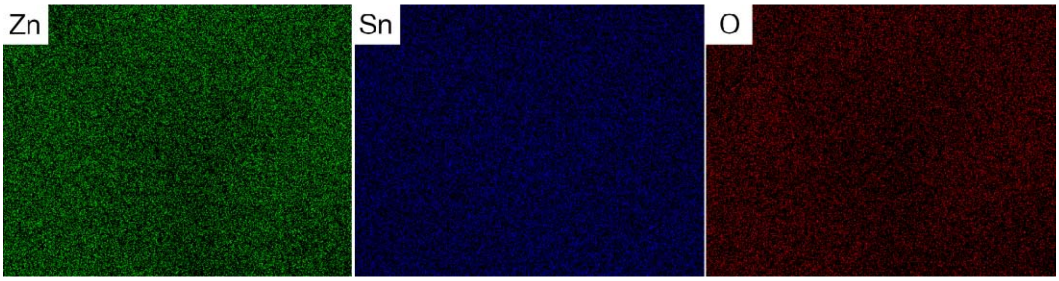

FIGURE S4 Corrsponding O, Sn and Zn elemental maps of the calcined product.

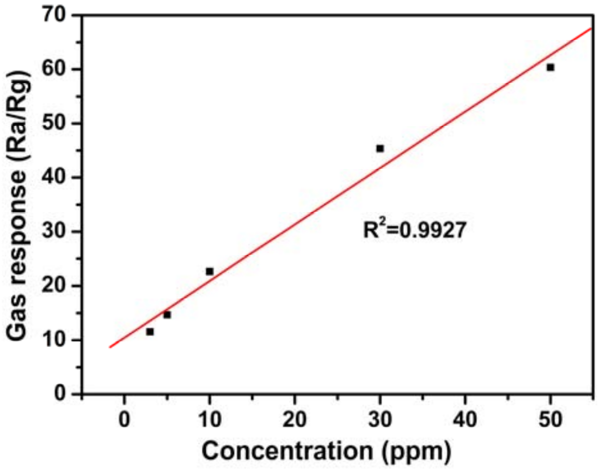

FIGURE S5 The relationship between the responses of Zn_2SnO_4 lamellar micro-spheres sensor and different concentrations of H_2S.

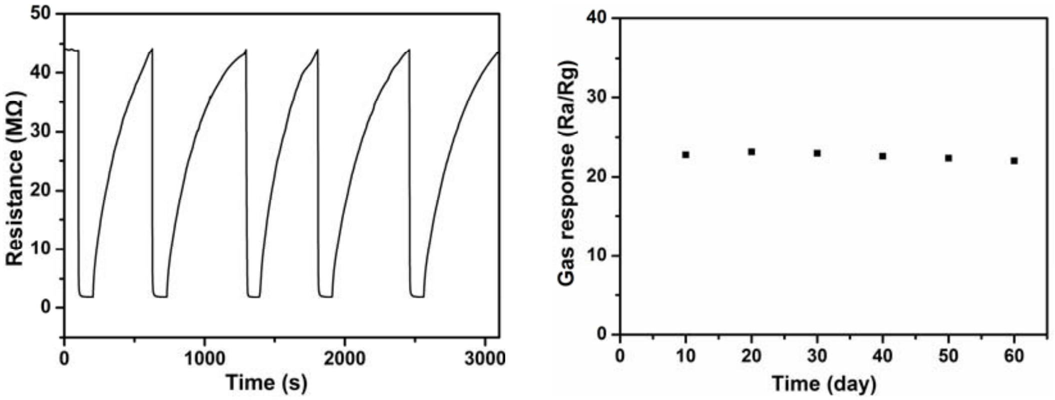

FIGURE S6 The reproducibility (a) and the long term stability (b) of the Zn_2SnO_4 sensor to 10 ppm H_2S measured at 170 °C.

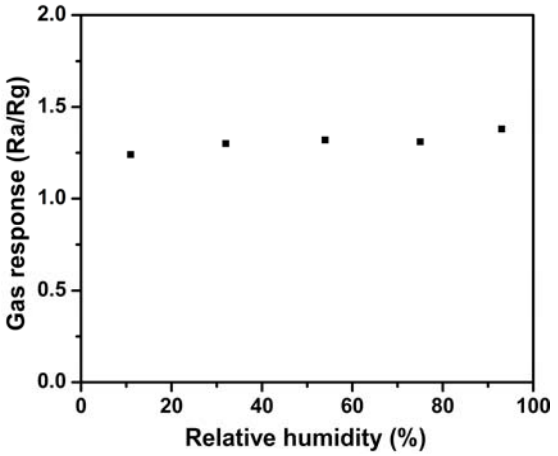

FIGURE S7 The responses of Zn_2SnO_4 sensor to different relative humidity atmospheres at 170 ^oC.

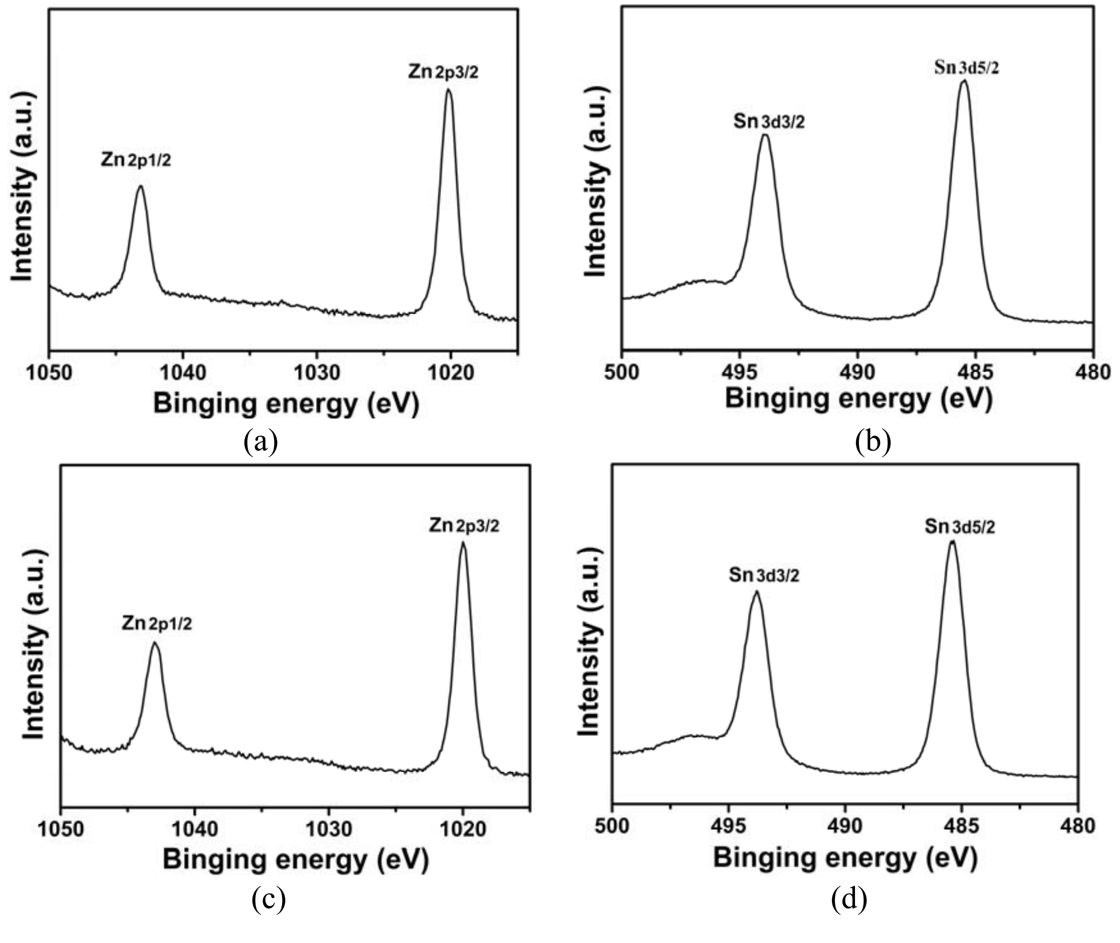

FIGURE S8 Zn 2p (a and c) and Sn 3d (b and d) XPS spectra of Zn_2SnO_4 sensor before (a and b) and after (c and d) the sensor exposure to H_2S at 170 ℃.

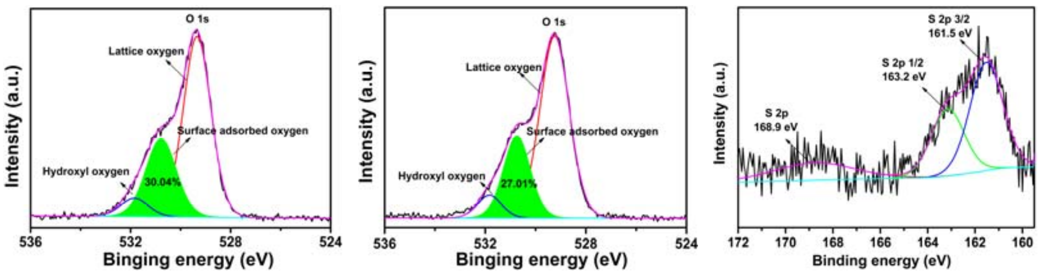

(a) (b) (c)

FIGURE S9 XPS spectra of Zn_2SnO_4 sensor before (a) and after (b and c) the sensor exposure to H_2S at 170 ℃.

REFERENCES

Sui, L. L., Xu, Y. M., Zhang, X. F., Cheng, X. L., Gao, S., Zhao, H., et al. (2015). Construction of three-dimensional flower-like α-MoO_3 with hierarchical structure for highly selective triethylamine sensor. *Sens. Actuators B* 208, 406–414. doi: 10.1016/j.snb.2014.10.138
